# Awareness and willingness to accept syphilis chemoprophylaxis among men who have sex with men from three cities in China: a cross-sectional study

**DOI:** 10.1186/s12889-022-14323-1

**Published:** 2022-10-15

**Authors:** Xu Zhang, Shu-Zhen Qi, Fang-Zhi Du, Zhi-Ju Zheng, Ning-Xiao Cao, Xiao-Li Zheng, Rui-Li Zhang, Qian-Qiu Wang

**Affiliations:** 1 Institute of Dermatology, Chinese Academy of Medical Science & Peking Union Medical College, National Center for STD Control, China Centers for Disease Control and Prevention, Nanjing, 210042 China; 2 Institute of Dermatology, Chinese Academy of Medical Science & Peking Union Medical College, Nanjing, 210042 China; 3 Department of Dermatology, The Second Affiliated Hospital of Nanjing Medical University, Nanjing, 210011 China

**Keywords:** Syphilis, MSM, Chemoprophylaxis, PrEP, PEP

## Abstract

**Background:**

The awareness and willingness to use doxycycline-based syphilis chemoprophylaxis among men who have sex with men (MSM) in China remain largely unknown.

**Methods:**

We recruited MSM online from Nanjing, Wuhan and Changsha between August and October of 2021, collected data from online survey, analyzed their data using descriptive statistics, and constructed binary logistic regression for factors associated with awareness and willingness to use chemoprophylaxis for syphilis and HIV.

**Results:**

Of 725 participants (44.0% of which resided in Nanjing, 37.7% in Changsha, and 18.3% in Wuhan), a majority were under 25 years of age; 62.2% had college degrees; 11.3% were HIV positive; and 5.10% had prior syphilis infection. Only 28.83% of participants had heard of syphilis chemoprophylaxis before. Odds of knowing syphilis chemoprophylaxis were higher in those who think it is necessary to have syphilis chemoprophylaxis versus those who think it is unnecessary (*P* = 0.002), and were higher in those whose acquaintance had chemoprophylaxis experience before (*P* < 0.001). Meanwhile, those who had no previous doxycycline using history, or had positive attitude were more likely to be willing to accept syphilis chemoprophylaxis (*P* = 0.009, *P* < 0.001). Over two-thirds (67.8%) of participants preferred the PEP mode in syphilis chemoprophylaxis, and side-effects of drugs remains their most worrying aspect.

**Conclusions:**

We observed elevated interest in syphilis chemoprophylaxis in MSM in our investigational areas, indicating that the combination of HIV and syphilis chemoprophylaxis in China is promising.

## Short summary

Our study found MSM in China had limited understanding of syphilis chemoprophylaxis, but they might be willing to accept syphilis PEP in the future.

## Introduction

There are growing epidemics of sexually transmitted infections (STIs) in China, many of which disproportionately affect men who have sex with men (MSM). The overall prevalence of HIV in China among MSM increased steadily from 2001 to 2018, an estimated 5.7% [1]. In addition, the prevalence of syphilis among MSM rose from 2.2 to 18.97% in recent 12 years, according to studies in different provinces in China [2–4].

Traditional preventive strategies are focused on long-term advertising and education; e.g. the promotion of consistent condom use, and frequent testing for HIV and STIs. These approaches to HIV and STI control may not be enough; thus, more effective and novel approaches such as antibiotic prophylaxis need to be evaluated and identified. Chemoprophylaxis such as pre-exposure prophylaxis (PrEP) has been proven to be effective in HIV prevention [5], limited evidence also showed that post-exposure prophylaxis (PEP) may be effective as well [6]. There is an ongoing multi-center trial among MSM (2018–2020, ChiCTR-IIN-17013762) in China that is concerned with the preventive agent *Truvada* for use in HIV prophylaxis. However, HIV PrEP may alter sexual interactions and condom-use patterns, unintentionally leading to an increased risk of bacterial STIs [7]. Therefore, it is critical to uncover new and effective STI-prevention strategies that will be acceptable to MSM; one feasible solution is to consider an analogous approach since HIV chemo-prophylactic technologies have achieved success in HIV prevention.

Only a few studies are focused on STI chemoprophylaxis at present. A pilot study of daily doxycycline administration as a syphilis PrEP in HIV-positive MSM in Los Angeles, USA, demonstrated a diminution in bacterial STIs compared with a contingency-management intervention [8]. Another study of doxycycline PEP among HIV-negative MSM comprising event-driven HIV PrEP in France found a 48% reduction in incident chlamydia, gonorrhea, and syphilis among MSM [9]. Recently, an on-going study in Seattle and San Francisco found taking doxycycline PEP within 72 h of condom-free sex sliced quarterly STI (including syphilis, chlamydia and gonorrhea) rates over 60% in HIV-positive MSM and transgender women (TGW) and in HIV-negative MSM and TGW taking PrEP against HIV [10]. Several other authors also assessed the current knowledge base, opinions, and the acceptability of STI-prevention techniques with respect to MSM. One study in California indicated that 67.5% of MSM community members were willing to use doxycycline to prevent syphilis or chlamydial infections if offered by their providers [11]. A study in Vancouver and Toronto also uncovered a considerable interest in syphilis chemoprophylaxis among MSM who attended local STI clinics [12]. Meanwhile, another study in Sydney found high-risk GBM (gay and bisexual men) preferred daily dosing regimen rather than event-driven regimen to prevent STIs [13]. To summarize, most of them were survey studies, and doxycycline PEP may be effective to STI prevention due to limited cohort study [9, 10].

Providing STI chemoprophylaxis as prevention in China may also be an alternative approach to addressing the increasing HIV and STI rates in MSM. Importantly, the researchers noted above were focused on participants from western, developed countries. However, considering the disparate cultures, behaviors, and transmission characteristics of STIs between developed countries and China, it is uncertain whether such chemo-prophylactic methods would work; and their effectiveness at prevention is also unknown. Thus, we herein generated a survey among MSM in three cities in China to evaluate their knowledge, willingness, and acceptability regarding chemoprophylaxis for prevention of syphilis and HIV; and considered whether it was feasible to add doxycycline to existing HIV chemoprophylaxis in China.

## Materials and methods

### Recruitment and data collection

We conducted a cross-sectional online survey among MSM from August 28 to October 30, 2021, in Nanjing, Changsha, and Wuhan, which are major cities in Eastern China, Southern China, and Central China, respectively.

MSM were recruited by local community-based organizations that employed a snowball sampling method to enroll participants with online platforms such as *WeChat*, *QQ*, *Blued* or other APPs. The online questionnaire was designed using the *Wenjuanxing* platform, which is the largest online questionnaire survey platform in China. All participants were provided with a QR code that they could scan using electronic equipment, enabling them to access the *Wenjuanxing* platform and to fill out the questionnaire. All survey results were only accessible to researchers.

### Survey methods

We collected the participants’ demographic characteristics—including nationality, age, marital status, monthly income, educational level, occupation, sexual orientation, HIV and syphilis test history, HIV status and prior syphilis infection.

Survey characteristics included awareness of HIV and Syphilis chemoprophylaxis (the survey included questions such as “have you ever heard of chemoprophylaxis?”; “do you know that Syphilis /HIV can be prevented with chemoprophylaxis?”; and “are people around you undergoing or have they undergone chemoprophylaxis?”; questions regarding their willingness or acceptability of chemoprophylaxis were “do you think it is necessary to take chemoprophylaxis for Syphilis/HIV?”; “can you accept chemoprophylaxis if the drugs are safe and freely provided?”; and “which type of chemoprophylaxis (PrEP or PEP) do you prefer?”).

Other characteristics such as numbers of sexual partners, frequency of condom use, and history of drug use were also collected for analysis. Surveys were administered exclusively online on the *Wenjuanxin* platform and designed to require less than five minutes to complete.

### Statistical analysis

Survey results were downloaded from online platforms and summarized in Office Excel 2016. We used descriptive statistics to depict demographic, behavioral, and sexually related baseline characteristics. To assess statistical differences of awareness and acceptability among various characteristic variables, we conducted Pearson’s *X*^2^ analysis, and when the results were significant (*P* < 0.05), we also executed a binary logistic regression analysis, and these significant factors were used as covariates. All analyses were conducted using R, version 3.5.2.

### Ethical approval and study funding

This study was approved by the Ethics Committee of the *Hospital for Skin Diseases, Institute of Dermatology, Chinese Academy of Medical Sciences, Peking Union Medical College* (2021-KY-034). The present study was funded by the *National Natural Science Foundation of China* (81772209).

## Results

### Participant characteristics

A total of 725 MSM were recruited to our survey, and of these, 319 were from Nanjing, 273 from Changsha, and 133 from Wuhan.

Nearly half of the participants were under 25 years of age (42.48%) and a majority were of Han ethnicity. Of the 725 participants, 62.21% had college degrees, 25.93% were students in a school, 80% were unmarried, 71.86% were homosexual, and 20.69% were bisexual; 68.11% had one to three sexual partners within one month, 56.11% had used a condom each time in their last five sexual encounters, and 5.66% exhibited a history of drug use; 86.48% had previously tested HIV, with 13.08% were HIV positive; 67.59% had been tested syphilis, with 5.10% had been infected with syphilis. Additional information regarding demographic and behavioral characteristics is shown in Table 1.

**Table 1:** Characteristics of MSM who participant in the study

Characteristics	Overall	(%)
**Nationality**		
Han	698	(96.28)
Other	27	(3.72)
**Age Group**		
< 25	308	(42.48)
26–35	232	(32.00)
36–45	105	(14.48)
> 45	80	(11.03)
**Educational level**		
Junior high school	39	(5.38)
High school	176	(24.28)
College	451	(62.21)
Postgraduate	59	(8.14)
**Marital status**		
Married	145	(20.00)
Unmarried	580	(80.00)
**Occupation**		
Student	188	(25.93)
Public institution	91	(12.55)
Private institution	185	(25.52)
Farmer	18	(2.48)
Freelancer	175	(24.14)
Other	68	(9.38)
**Monthly Income (CNY)**		
< 2000	180	(24.83)
2000–5000	180	(24.83)
5000–8000	189	(26.07)
8000–12,000	116	(16.00)
> 12,000	60	(8.28)
**Sexual orientation**		
Homosexual	521	(71.86)
Bisexual	150	(20.69)
Other^a^	54	(7.45)
**Role in homosexual behavior in recent 1 year**		
Receptive	229	(31.59)
Insertive	239	(32.97)
Other^b^	257	(35.45)
**Sexual partners in one month**^**c**^		
None	200	(27.97)
1–3	487	(68.11)
> 3	28	(3.92)
**Number of times using a condom in the last five sexual encounters**^**d**^		
0	107	(14.86)
1	53	(7.36)
2	40	(5.56)
3	62	(8.61)
4	54	(7.50)
≥ 5	404	(56.11)
**Previous hard-drug use**		
Yes	41	(5.66)
No	684	(94.34)
**Previous use of HIV prophylaxis medications**		
Yes	50	(6.90)
No	675	(93.10)
**HIV tested**		
Yes	627	(86.48)
No	98	(13.52)
**Syphilis tested**		
Yes	490	(67.59)
No	235	(32.41)
**HIV status**		
Positive	82	(13.08)
Negative	545	(86.92)
**Syphilis infected**		
Yes	37	(7.55)
No	453	(92.45)

^a^Includes those who also have other sexual orientations, e.g. *Pansexual*

^b^Those who played both receptive and insertive roles in previous homosexual behaviors

^c^Ten participants produced invalid results

^d^Five participants produced invalid results

### Awareness and associated characteristics

Up to 520 participants (71.72%) had previously heard of PrEP or PEP prior to completing the questionnaire, and of these, 471 (64.97%) knew about HIV chemoprophylaxis; but only 209 (28.83%) knew about syphilis chemoprophylaxis (Table 2). As shown in Table 3, awareness of syphilis chemoprophylaxis was significantly associated with HIV testing history, syphilis infection status, whether people around are undergoing or have undergone chemoprophylaxis, previous use of doxycycline, previous use of HIV prophylaxis medications and whether they think it is necessary for syphilis chemoprophylaxis (Table 3, *P* < 0.05).

**Table 2:** Awareness and willingness of participants towards chemoprophylaxis

**Characteristics**	**Overall**	(**%**)
**Heard of chemoprophylaxis before**		
Yes	520	71.72
No	205	28.28
**Heard of HIV chemoprophylaxis before**		
Yes	471	64.97
No	254	35.03
**Heard of syphilis chemoprophylaxis before**		
Yes	209	28.83
No	516	71.17
**People around are undergoing or have undergone chemoprophylaxis?**		
Yes	294	40.55
Not sure	373	51.45
No	58	8.00
**Willing to undergo chemoprophylaxis**		
Yes, only for HIV	93	12.83
Yes, only for syphilis	22	3.03
Yes, for both HIV and syphilis	542	74.76
No	68	9.38

**Table 3:** Characteristics associated with awareness to syphilis chemoprophylaxis among participants

**Characteristics**	**Heard of syphilis chemoprophylaxis**		**Not heard of syphilis chemoprophylaxis**		*P*-value^*^	aOR (95% CI)^**^	a*P-*value**
	**n**	**(%)**	**n**	**(%)**			
**HIV tested**							
Yes	193	30.78%	434	69.22%	0.005		0.060
No	16	16.33%	82	83.67%			
**Syphilis infected**							
Positive	5	13.51%	32	86.49%	0.031		0.601
Negative	145	32.01%	308	67.99%			
**People around are undergoing or have undergone chemoprophylaxis?**							
Yes	118	40.14%	176	59.86%	< 0.001		**< 0.001**
Not sure	80	21.45%	293	78.55%			
No	11	18.97%	47	81.03%			
**Previous use of Doxycycline**							
Yes	14	51.85%	13	48.15%	0.013		0.066
No	195	27.94%	503	72.06%			
**Previous use of HIV prophylaxis medications**							
Yes	22	44.00%	28	56.00%	0.022		0.344
No	187	27.70%	488	72.30%			
**Think it is necessary for syphilis chemoprophylaxis**							
Yes	192	31.63%	415	68.37%	< 0.001	Ref	**0.002**
No	17	14.41%	101	85.59%		0.870 (0.796–0.950)	

^*^Based on Pearson’s χ2 test

^**^Based on binary logistic regression analysis

After adjusting for the aforementioned variables with binary logistic regression analysis, awareness of syphilis chemoprophylaxis was still significant with whether people around are undergoing or have undergone chemoprophylaxis (*P* < 0.001) and whether think it is necessary for syphilis chemoprophylaxis (Table 3, *P* = 0.002).

### Willingness and associated characteristics

In this survey, we asked participants whether they will accept chemoprophylaxis if the drugs are safe and freely provided, and as a result, up to 657 MSM (90.6%) were willing to accept chemoprophylaxis. Among them, 542 were willing to accept both HIV and syphilis chemoprophylaxis, 22 were willing to accept syphilis prophylaxis solely (Table 2). Using Pearson’s χ^2^ analysis, we identified several characteristics associated with willingness to syphilis chemoprophylaxis: educational level, sexual orientation, previous hard-drug use history, syphilis testing history, previous use of doxycycline, whether they had heard of HIV chemoprophylaxis before and whether they think it is necessary for syphilis or HIV chemoprophylaxis (Table 4, *P *< 0.05).

**Table 4:** Characteristics associated with willingness to undergo syphilis chemoprophylaxis among participants

**Characteristics**	**Willing to undergo syphilis chemoprophylaxis**		**Unwilling to undergo syphilis chemoprophylaxis**		*P*-value*	aOR (95% CI)**	a*P*-value**
	**n**	**(%)**	**n**	**(%)**			
**Educational level**							
Junior high school	25	64.10%	14	35.90%	0.048		0.182
High School	134	76.14%	42	23.86%			
College	363	80.49%	88	19.51%			
Postgraduate	42	71.19%	17	28.81%			
**Sexual orientation**							
Homosexual	420	80.61%	101	19.39%	0.013		0.090
Bisexual	105	70.00%	45	30.00%			
Other^a^	39	72.22%	15	27.78%			
**Previous hard-drug use**							
Yes	37	90.24%	4	9.76%	0.001		0.106
No	257	62.08%	157	37.92%			
**Syphilis tested**							
Yes	397	81.02%	93	18.98%	0.003		0.108
No	167	71.06%	68	28.94%			
**Previous use of Doxycycline**							
Yes	16	59.26%	11	40.74%	0.034	Ref	**0.009**
No	548	78.51%	150	21.49%		1.228 (1.054–1.430)	
**Heard of HIVchemoprophylaxis before**							
Yes	380	80.68%	91	19.32%	0.014		0.680
No	184	72.44%	70	27.56%			
**Think it is necessary for HIV chemoprophylaxis**							
Yes	499	82.34%	107	17.66%	< 0.001		0.155
No	65	54.62%	54	45.38%			
**Think it is necessary for syphilis chemoprophylaxis**							
Yes	504	83.03%	103	16.97%	< 0.001	Ref	**< 0.001**
No	60	50.85%	58	49.15%		0.783 (0.698–0.879)	

^*^Based on Pearson’s χ2 test

^**^Based on binary logistic regression analysis

^a^Includes those who also have other sexual orientations, e.g. *Pansexual*

After controlling for the above significant variables in a binary logistic regression analysis, MSM who thought it was unnecessary for syphilis chemoprophylaxis (aOR = 0.783, 95% CI = 0.698–0.879) or had used doxycycline before (aOR = 0.814, 95% CI = 0.699–0.949) were significantly less likely to accept syphilis chemoprophylaxis, compared with those men who had tested for syphilis previously or had not used doxycycline before (Table 4).

### Willingness to use different forms of chemoprophylaxis

Most participants were willing to use syphilis PEP relative to PrEP (67.9% vs. 32.1%, respectively), and they responded similarly with respect to HIV PEP and PrEP (68.4% vs. 31.6%, respectively). Those who had not heard of PrEP/PEP before were more likely to choose syphilis PrEP than PEP after binary logistic regression analysis (aOR = 1.205, 95% CI = 1.109–1.309), and those who had not undergone PrEP/PEP previously were less likely to choose syphilis PrEP than PEP (aOR = 0.863, 95% CI = 0.750–0.992) (Table 5). In contradistinction, monthly income was found to be significantly associated with willingness to choose HIV PrEP or PEP after binary logistic regression analysis (*P* = 0.01). Furthermore, those who had not taken PrEP/PEP before were less likely to choose HIV PrEP than PEP compared with those who had experienced PrEP/PEP (aOR = 0.817, 95% CI = 0.724–0.922) (Table 6).

**Table 5:** Characteristics associated with willingness to undergo syphilis PrEP and syphilis PEP

**Characteristics**	**Prefer to Syphilis PrEP**		**Prefer to Syphilis PEP**		*P*-value*	aOR (95% CI)**	a*P*-value**
	**n**	**(%)**	**n**	**(%)**			
**Sexual partners in one month**^**a**^							
None	75	37.5	125	62.5	0.024		0.892
1–3	149	30.6	338	69.4			
> 3	4	14.29	24	85.71			
**HIV status**							
Positive	36	43.9	46	56.1	0.019		0.309
Negative	165	30.28	380	69.72			
**Heard of chemoprophylaxis before**							
Yes	146	28.08	374	71.92	< 0.001	Ref	** < 0.001**
No	87	42.44	118	57.56		1.205 (1.109–1.309)	
**Previous chemoprophylaxis undertake**							
Yes	32	48.48	34	51.52	0.004	Ref	**0.038**
No	201	30.5	458	69.5		0.863 (0.750–0.992)	

^*^Based on Pearson’s χ2 test

^**^Based on binary logistic regression analysis

^a^Ten participants produced invalid results

**Table 6:** Characteristics associated with willingness to undergo HIV PrEP and HIV PEP

**Characteristics**		**Prefer to HIV PrEP**		**Prefer to HIV PEP**		*P*-value*	aOR (95% CI)**	a*P*-value**
		**n**	**(%)**	**n**	**(%)**			
**Occupation**								
Student		66	35.11	122	64.89	< 0.001	—	0.383
Public institution		24	26.37	67	73.63			
Private institution		55	29.73	130	70.27			
Farmer		5	27.78	13	72.22			
Freelancer		55	31.43	120	68.57			
Other		24	35.29	44	64.71			
**Monthly Income (CNY)**								
< 2000		61	33.89	119	66.11	0.028	—	**0.01**
2000–5000		71	39.44	109	60.56			
5000–8000		50	26.46	139	73.54			
8000–12,000		34	29.31	82	70.69			
> 12,000		13	21.67	47	78.33			
**HIV status**								
Positive		35	42.68	47	57.32	0.026	—	0.388
Negative		162	29.72	383	70.28			
**Previous chemoprophylaxis undertake**								
Yes	34		51.52	32	48.48	< 0.001	Ref	**0.001**
No	195		29.59	464	70.41		0.817 (0.724–0.922)	

^*^Based on Pearson’s χ2 test

^**^Based on binary logistic regression analysis

### Concerns regarding chemoprophylaxis

**Fig. 1:**
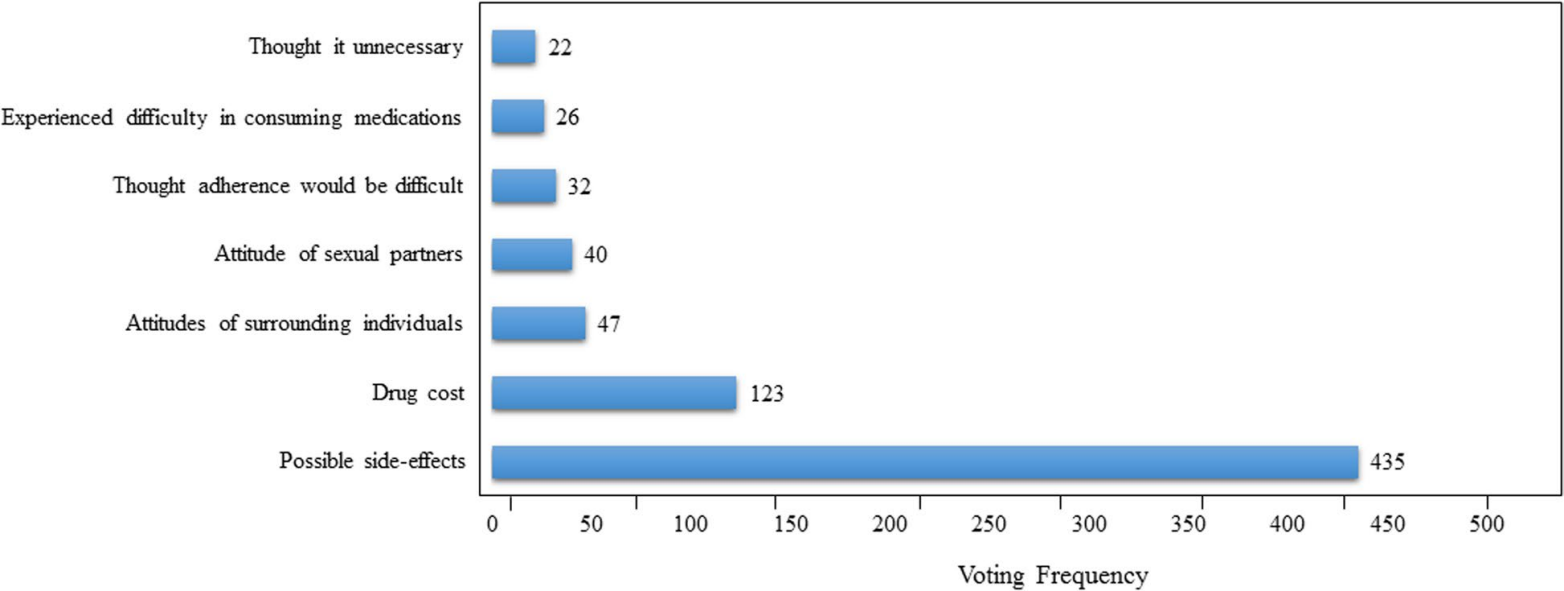
Primary concerns regarding chemoprophylaxis among participants

Among the 725 participants, a majority chose “side-effects” as their primary concern regarding chemoprophylaxis (60.0%). Other primary concerns included drug price (17.0%), attitudes of others (12.0%), think adherence or consume medications would be difficult (8.0%), and no necessity (3.0%). Further information is provided in Fig. 1.

## Discussion

We surveyed MSM participants recruited by local social workgroups from three cities in China to assess their awareness and willingness to use chemoprophylaxis. Our data suggested that most MSM had heard of HIV chemoprophylaxis before, but only a few of them had heard of syphilis chemoprophylaxis. Chemoprophylaxis appeared to be highly accepted if it was safe and freely provided, and we found that most of the MSM preferred PEP over PrEP, both in HIV and syphilis chemoprophylaxis. In addition, we realized that drug side-effects constituted the major barrier to acceptance of chemoprophylaxis by MSM. Syphilis chemoprophylaxis with doxycycline is a novel strategy that has not yet gained wide acceptance. To our knowledge, this is the first study to explore awareness and willingness in both syphilis and HIV chemoprophylaxis among MSM in China.

The awareness of syphilis chemoprophylaxis was quite low, compared with HIV chemoprophylaxis (28.83% vs. 64.97%). In fact, the chemoprophylaxis for STIs remains a brand-new concept, compared with HIV chemoprophylaxis. Therefore, it is necessary to strengthen science popularization and education among MSM in China, introducing up-to-date STI control or prevention technologies towards them. We found that those who think it is necessary for syphilis chemoprophylaxis were more likely to heard of syphilis chemoprophylaxis before, and it agreed with previous study that concerns about contracting STI will promote MSM to seek for more prevention measures towards STI [11]. Interestingly, we found that if one’s friends or associates were undergoing or had undergone chemoprophylaxis, then this individual would be more likely to know about PrEP/PEP as well. As a result, imparting knowledge of chemoprophylaxis to certain individuals could help to address additional people indirectly, and thus contribute to community connections.

Notably, willingness to use syphilis chemoprophylaxis was as high as 78% and was higher than most of results shown in previous studies, as other authors reported acceptability rates ranging from 40 to 80% [12, 14, 15]. Importantly, this acceptance rate was based on the premise that preventive drugs are safe and free of charge; thus, MSM were willing to accept chemoprophylaxis if barriers could be removed, as side-effects and drug costs comprised the top two barriers according to our investigation. In addition, individuals who had used doxycycline before were significantly less likely to accept syphilis chemoprophylaxis, suggesting that they may had side-effects after using doxycycline before. Hopefully, the doxycycline enteric-coated capsule has less side-effects compared with traditional doxycycline pills and may be the appropriate choice in chemoprophylaxis in the future [16, 17]. Besides, recent studies found that there were no patients (who had syphilis or chlamydia trachomatis infection) with treatment failure due to doxycycline antimicrobial resistance during long period of treatment [18, 19]. It suggested that long-time chemoprophylaxis with doxycycline may be safe as well, but still need more evidence to prove it.

Participants’ willingness to accept PEP was considerably higher than to accept PrEP in our study. This result was similar to the prior studies in Toronto (willingness to use syphilis PrEP vs. syphilis PEP, 42.3% and 66.9%, respectively; and willingness to use HIV PrEP vs. HIV PEP, 87.7% and 91.0%, respectively) and Vancouver (willingness to use syphilis PrEP vs. syphilis PEP, 46.5% and 51.4%, respectively) [12]. Such results are reasonable since accepting PrEP entails higher economic costs and additional pills to be taken by MSM. Intriguingly, only those who had PrEP/PEP experience previously were more likely to choose PrEP in both HIV and syphilis chemoprophylaxis. A small Australian study had showed that high-risk GBM preferred daily STI PrEP than PEP [13]. We posit that people with PrEP/PEP experience are more likely to develop the habit of taking treatment drugs in a timely fashion; however, to those who are new to chemoprophylaxis, PEP may be the preferred choice.

Our study was subject to several limitations. First, the participants were enrolled via a local social group and all the surveys were conducted online, which could have caused recruitment bias. Second, the responses to questionnaires were self-reported by participants, and this may have led to social-desirability bias and recall bias. Third, our investigation entailed a cross-sectional design, making it difficult to perceive causal relationships. Therefore, we used concomitant variables in our correlation analysis to reduce these impacts. Fourth, we asked participants’ willingness to take prevention drugs under the assumption that drugs are safe and freely provided, which may increase their motivations. Fifth, we didn’t show the efficacy differences between PrEP and PEP to participants. Most published studies showed high efficacy of HIV PrEP and syphilis PEP only, but lack evidence for HIV PEP and syphilis PrEP, and it may be a factor that will affect their choses. Lastly, only three cities were chosen for our survey, and the results may thus not have been representative of the overall situation in China.

Despite the aforementioned limitations, to our knowledge, this is the first study to investigate the awareness of and willingness toward syphilis chemoprophylaxis among Chinese MSM. We found willingness towards syphilis chemoprophylaxis were higher than expected, indicating the possibility of promotion. New approaches to resolving STIs such as syphilis are needed since the chemoprophylaxis for HIV has provided a favorable modality for reference, and it may be possible to combine these two preventive strategies together. Although the widespread promotion of syphilis chemoprophylaxis should be based on more robust data regarding the safety and cost-effectiveness of drugs, we posit that this strategy may constitute a promising and novel direction for STIs prevention in the future.

## Data Availability

The datasets used and/or analyzed during the current study are available from the corresponding author on reasonable request.
